# Neo-Sinus Washout Time Following Transcatheter Aortic Valve Replacement and Hemodynamic Outcomes

**DOI:** 10.1016/j.shj.2025.100686

**Published:** 2025-06-21

**Authors:** Shivabalan Kathavarayan Ramu, Toshiaki Isogai, Saksham Beotra, Vishwum Kapadia, Nikita Thakore, Ankit Agrawal, Shashank Shekhar, Maryam Muhammad Ali Majeed-Saidan, Rohan Prasad, Agam Bansal, Abdelrahman Abushouk, Odette Iskandar, Larisa G. Tereshchenko, Grant Reed, Rishi Puri, James Yun, Serge Harb, Rhonda Miyasaka, Zoran Popovic, Amar Krishnaswamy, Samir R. Kapadia

**Affiliations:** a Department of Cardiovascular Medicine, Heart, Vascular and Thoracic Institute, Cleveland, Ohio, USA; b Department of Quantitative Health Sciences, Lerner Research Institute, Cleveland Clinic, Cleveland, Ohio, USA

**Keywords:** Bioprosthetic valve dysfunction, Neo-sinus washout time, Transcatheter aortic valve replacement

## Abstract

**Background:**

Leaflet thrombosis and transcatheter heart valve dysfunction are key concerns following transcatheter aortic valve replacement (TAVR). Prolonged neo-sinus washout time (NWT) may predispose patients to hypoattenuated leaflet thickening (HALT) and leaflet thrombosis, increasing the risk of valve degeneration. This study evaluates the association between in vivo NWT derived from aortograms using computer vision and hemodynamic outcomes at 30 days and 1 year post-TAVR.

**Methods:**

We retrospectively analyzed 2254 patients (mean age: 79 ​± ​9 years, 40% female) who underwent TAVR with balloon-expandable valves between 2016 and 2020. Patients were tertile stratified into tertile 1 (T1) (1.444-1.870 ​s; n ​= ​752), T2 (1.870-1.939 ​s; n ​= ​752), and T3 (1.939-2.110 ​s; n ​= ​751) based on their NWTs.

**Results:**

At 30 days, T3 had a higher transvalvular aortic valve mean gradient than T1 (12.61 ​± ​5.07 mmHg vs. 11.98 ​± ​4.75 mmHg, *p* ​= ​0.03). In multivariate modeling, T3 was significantly associated with increased aortic valve mean gradient compared to T1 (estimate: 0.703, *p* ​= ​0.02). NWT was higher in patients with HALT at 30 days (1.970 ​± ​0.047 ​s vs. 1.889 ​± ​0.100 ​s, *p* ​= ​0.001), with each 0.1 ​second increase in NWT tripling the odds of HALT.

**Conclusions:**

Prolonged NWT is associated with higher transvalvular gradients and independently predicts HALT post-TAVR. NWT may serve as a novel marker to identify patients at risk of valve dysfunction and guide early pharmacotherapy.

## Highlights

• Retrospective analysis of 4D computed tomography data from the Assessment of Transcatheter and Surgical Aortic Bioprosthetic Valve Thrombosis and Its Treatment with Anticoagulation (RESOLVE) trial has revealed that one of the potential mechanisms of transcatheter heart valve thrombosis is neo-sinus flow stasis.
• The time taken for contrast material to clear from the neo-sinus in vivo is quantified as the neo-sinus washout time (NWT).
• The application of a computer vision algorithm to quantify NWT immediately postprocedure presents a novel tool for identifying patients at risk of early valve deterioration and for guiding postprocedural pharmacotherapy.
• In a cohort study of 2254 patients who underwent transcatheter aortic valve replacement (TAVR), those with prolonged NWT—indicated by slower clearance of dye particles in the algorithm-marked frame—demonstrated higher transvalvular aortic mean gradients at 30-day and 1-year follow-ups. Furthermore, NWT showed high predictive accuracy for 30-day hypoattenuated leaflet thickening (HALT) post-TAVR.
• Prolonged NWT, as identified by a computer vision algorithm, may serve as a significant predictor of HALT and elevated gradients post-TAVR.

## Introduction

Transcatheter aortic valve replacement (TAVR) has revolutionized the treatment of severe aortic stenosis, yet bioprosthetic valve thrombosis remains a notable concern, occurring in both subclinical and clinical forms.^1^, ^2^, ^3^, ^4^ Subclinical leaflet thrombosis (SLT) is characterized by hypoattenuated leaflet thickening (HALT) with or without reduced leaflet motion (RELM), whereas clinical leaflet thrombosis (CLT) manifests as hemodynamic deterioration and symptomatic valve dysfunction.^2^, ^3^, ^4^, ^5^, ^6^ The hemodynamic consequences of leaflet thrombosis include increased transvalvular pressure gradients, reduced valve leaflet mobility, and, in severe cases, structural valve degeneration, potentially leading to valve failure.^2^^,^^7^, ^8^, ^9^ While SLT alone may not always result in immediate adverse events, its progression to CLT has been associated with an increased risk of stroke.^10^^,^^11^

In vitro studies have attempted to provide mechanistic insights into the interplay between patient- and procedure-related factors that might predispose to leaflet thrombosis.^12^, ^13^, ^14^, ^15^, ^16^ Meanwhile, obesity, valve-in-valve TAVR, male sex, larger valve sizes, and larger sinus of Valsalva were associated with CLT.^2^ Additionally, the phenomenon of neo-sinus stasis—the stagnation of blood within the three-dimensional space between the native and transcatheter heart valve (THV) leaflets—has been identified as a key contributor to thrombosis in computational and in vitro studies.^7^^,^^16^, ^17^, ^18^ While blood stasis in the neo-sinus has been suggested as a potential mechanism for leaflet thrombosis, conclusive clinical evidence supporting this assertion remains limited.^15^^,^^19^ Computer vision is a subfield of artificial intelligence (AI) enabling computers to derive information from images, videos, and related visual inputs.^20^ This technology has the capability to track and quantify the movement of objects within a given frame over time, making it a powerful tool for analyzing fluid dynamics in medical imaging.^21^ Given the challenges of routine 4-dimensional (4D) computed tomography (CT) implementation in clinical practice, AI-driven image analysis could facilitate earlier detection of prolonged contrast washout within the neo-sinus—a potential indicator of future leaflet thrombosis.

We hypothesize that patients with prolonged washout time of contrast dye within the neo-sinus are predisposed to a future risk of THV dysfunction and HALT. In this study, we aimed to retrospectively quantify the neo-sinus washout time (NWT) in aortogram sequences following TAVR valve deployment using computer vision and determine the association between NWT and THV dysfunction. By leveraging AI-based image processing, we aim to establish NWT as a potential early marker for predicting leaflet thrombosis, ultimately improving patient outcomes through earlier intervention.

## Methods

### Study Population

**Figure 1:**
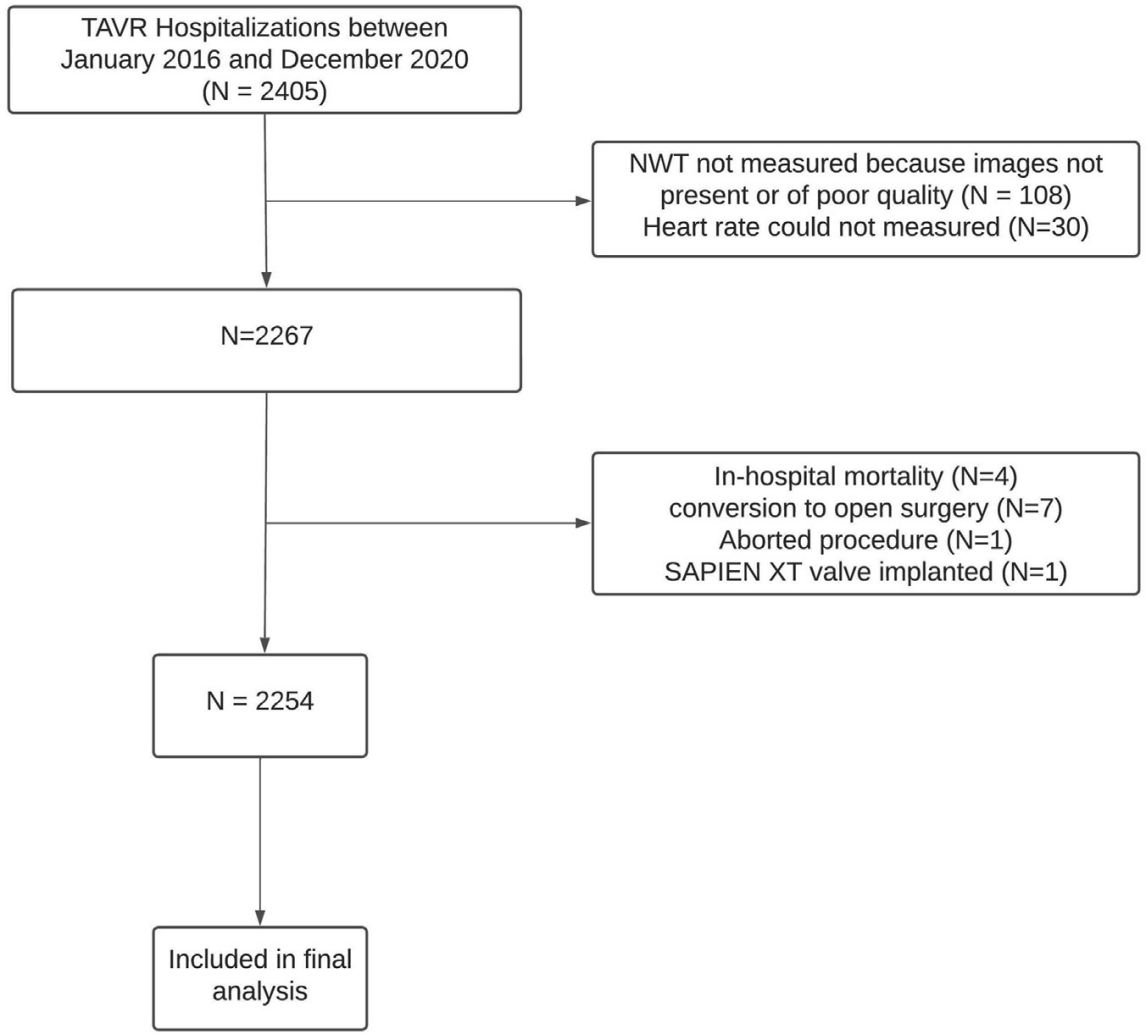
Study workflow. Workflow showing patient exclusion criteria and how the final study cohort was reached. Abbreviations: NWT, neo-sinus washout time; TAVR, transcatheter aortic valve replacement.

We conducted a retrospective cohort study on consecutive patients who underwent TAVR with balloon-expandable valves between January 2016 and December 2020 at the Cleveland Clinic, Ohio. TAVR procedures were performed with Edwards SAPIEN XT and SAPIEN 3 valves (Edwards Lifesciences) and S3 Ultra (Edwards Lifesciences). The study protocol was approved by the institutional review board at the Cleveland Clinic. We included adult patients with aortic stenosis who underwent TAVR. Additionally, we excluded patients whose heart rate (HR) could not be measured, those who experienced in-hospital mortality, converted to open surgery, and had the procedure aborted. The study workflow is summarized in Figure 1.

#### Measurement of Neo-Sinus Washout Time

**Figure 2:**
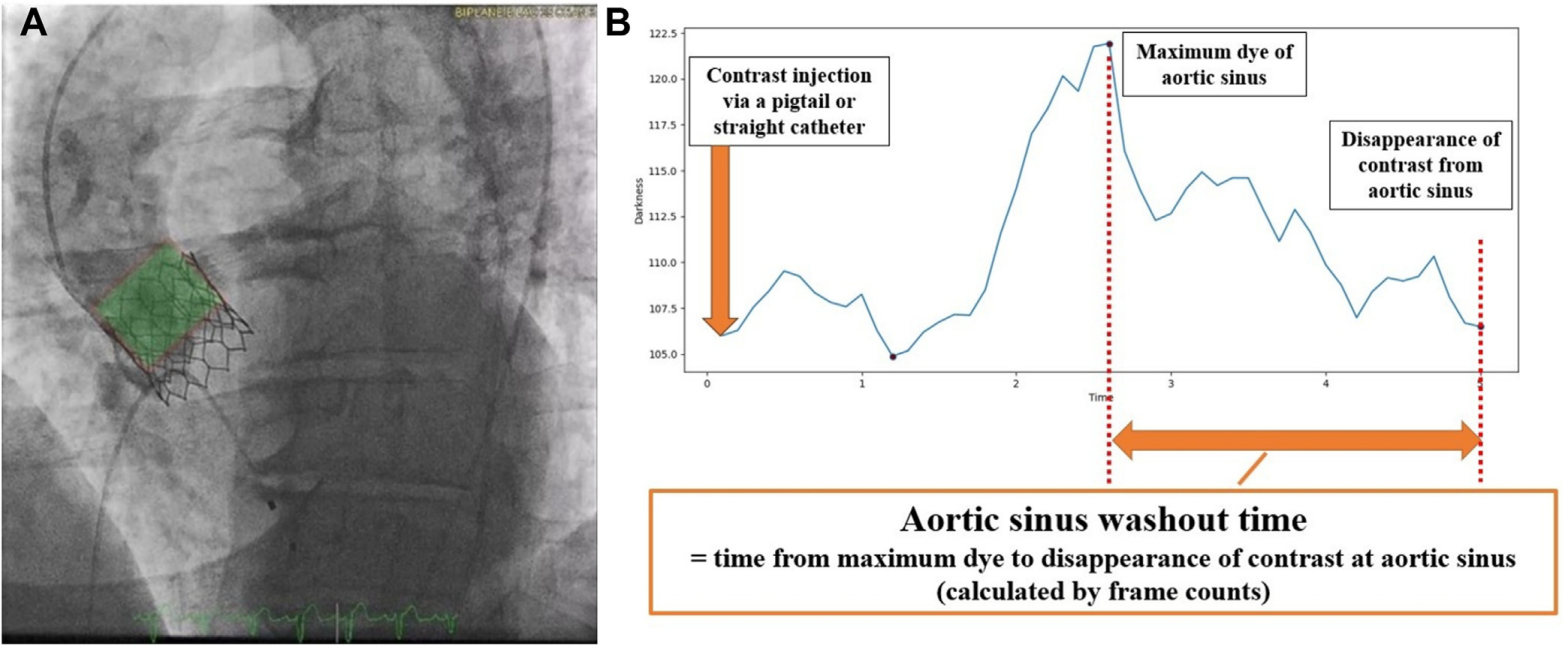
Method of neo-sinus washout time measurement. Rectangular marker (red) placed over the stent in post-TAVR angiogram (a). Schematic diagram showing the amount of contrast material over time for a given sequence. The time taken for the maximum amount of detected contrast to disappear is measured as washout time (b). TAVR, transcatheter aortic valve replacement.

One crucial step in the TAVR procedure is the routinely performed postimplantation aortography. It facilitates the assessment of coronary flow, confirmation of the depth of implantation, and detection of aortic regurgitation post-TAVR procedure. During this step, the contrast dye is injected at a rate of 20 cc/sec rate over 1 ​second, with a total volume of 20 cc, into the aortic root. The contrast washout is indicated by the gradual disappearance of the opacification observed in the aorta and left ventricle. While the washout of dye from the areas outside the frame of the valve in the sinuses corresponds to the washout of the native aortic sinuses, the washout within the frame is considered as the washout of the dye from the sinuses of the implanted valves or neo-sinus. Aortography sequences were examined using Syngo Dynamics software (Siemens Medical Solutions Inc, USA) where the images stored at 512 ​× ​512 dpi resolution. Each sequence consists of multiple frames, the number of which depends on the frame rate of the event. Measurement of NWT was performed with a custom Python **OpenCV** script. OpenCV is an advanced computer vision library used for image processing, object detection, and motion tracking. In the first frame of a postimplantation aortogram sequence, a rectangular marker was manually positioned along the borders of the stent. A subjective cutoff of 20% of the space was left above the upper border of the stent (Figure 2a) to allow for the measurement of any contrast material refluxing beyond the stent’s upper border during dye injection. OpenCV then tracked the valve across the subsequent frames, using the manually selected first frame as a reference. The pixel intensity within each marked box was measured frame by frame over time again using OpenCV. The lower the pixel intensity, the greater is the darkness value (0 [completely dark] to 255 [completely bright]), which correlates with the presence of the contrast material. Subsequently, using Python, the maximum intensity value within the sequence was identified, followed by the minimum intensity value occurring after the maximum. The difference between these values provided the washout time (in milliseconds, later converted to seconds), which was recorded as NWT (Figure 2b). In cases where balloon postdilation was performed, the AI-based algorithm analyzed the postdilation sequence, ensuring that NWT was measured only after postdilation.

The HR-adjusted NWT was calculated by an equation derived from linear regression to adjust for confounders during measurement as: Adjusted NWT ​= ​2.12 + 0.00254⋅First Angle Value + 0.0036⋅Second Angle Value−0.00476⋅Heart Rate, expressed in seconds. Heart rate is the rate during the sequence at which the NWT is measured; first and second angle values are the fluoroscopic projection angles at the right anterior oblique/left anterior oblique view and cranial/caudal views, respectively. The model as a whole explains a small portion of the variance in NWT (1.27%). Among the variables, HR has the largest individual contribution, explaining 0.53% of the variance, while First Angle Value and Second Angle Value make smaller contributions, explaining 0.11 and 0.12% of the variance, respectively.

### Outcomes

The primary outcomes included longitudinal changes in hemodynamic parameters (aortic valve mean gradient [AVMG] and dimensionless valve index [DVI]) immediately post-TAVR, at 30 days, and at 1 year following TAVR, stratified by NWT. Secondary outcomes involved determining the predictive value of NWT in detecting HALT during follow-up.

### Statistical Analysis

Categorical variables were reported as frequencies and percentages. Continuous variables were presented as mean and SD or median and interquartile range (IQR). To test for trend in binomial proportions across the levels of a washout time tertiles, the Cochran Armitage test was conducted. Similarly, to test for trend in continuous variables, the Jonckheere-Terpstra test, which is a nonparametric test for ordered differences among classes was conducted. To compare the hemodynamic outcomes between tertiles, the analysis of variance test was used. Tukey’s corrected *p* values were calculated for comparisons between the first and third tertiles. The Mann-Whitney test was used to compare the NWT between patients who had HALT and those who did not at different follow-up times. Linear generalized estimating equations (GEE) models were used to fit population-averaged generalized linear models, distinct for each outcome (AVMG and DVI).^16^ A complete case analysis approach was used to fit GEE models. Missing follow-up outcome data were not imputed and were considered to be missing at random. We used the GEE approach to account for the repeated measures of echocardiographic outcomes over time. The within-patient correlation structure was estimated as autoregressive of order 1. The canonical link function for the Gaussian family was used. A two-tailed *p* value of <0.05 was set to be statistically significant. Firth bias-adjusted logistic regression was used to account for the small event size of HALT and to find predictors of the same. Statistical analyses were conducted using Python programming language version 3.6 (Python Software Foundation).

## Results

The baseline and procedural characteristics of patients, stratified by tertile according to HR-adjusted NWT, are shown in Table 1. The mean unadjusted NWT is 1.89 ​seconds with a relatively wide 95% CI (1.85-1.93 ​seconds), indicating some variability in the data. The SD of 0.89 ​seconds and a range of 6.2 ​seconds (minimum: 0.1, maximum: 6.3) show a notable dispersion of values. After adjustment, the mean NWT becomes 1.8897 ​seconds, with a much narrower 95% CI (1.88-1.89 ​seconds). The corrected NWT exhibits a negative skewness of −1.438, indicating a left-tailed distribution.

**Table 1:** Baseline and procedural characteristics

Characteristic	No. (%)		
	HR adjusted neo-sinus washout time T1 (1.4442–1.8696 ​s) (n ​= ​751)	HR adjusted neo-sinus washout time T2 (1.8697–1.9388 ​s) (n ​= ​752)	HR adjusted neo-sinus washout time T3 (1.9388–2.1095 ​s) (n ​= ​751)
Age, mean, y	78.43 ​± ​9.23	79.32 ​± ​8.97	79.56 ​± ​9.03
Female sex	317 (42.2%)	319 (42.4%)	278 (37.0%)
Bicuspid native aortic valve	47 (6.2%)	42 (5.6%)	33 (4.4%)
Height, cm	170.57 ​± ​59.81	170.58 ​± ​72.37	168.94 ​± ​11.35
Weight. kg	84.67 ​± ​22.28	83.20 ​± ​22.17	82.63 ​± ​19.82
BMI, kg/m^2^	38.23 ​± ​226.34	30.19 ​± ​19.81	29.36 ​± ​15.86
BSA by Mosteller method, m^2^	1.98 ​± ​0.34	1.96 ​± ​0.33	1.96 ​± ​0.26
NYHA class III/IV	591 (78.6%)	568 (75.5%)	547 (72.8%)
STS risk score	6.29 ​± ​4.55	5.94 ​± ​4.37	5.52 ​± ​3.71
Hypertension	671 (89.2%)	681 (90.6%)	681 (90.7%)
Diabetes mellitus	319 (42.4%)	272 (36.2%)	252 (33.6%)
Current dialysis	19 (2.5%)	26 (3.5%)	23 (3.1%)
Coronary artery disease	406 (54.0%)	389 (51.7%)	389 (51.8%)
History of atrial fibrillation or flutter	331 (44.0%)	307 (40.8%)	331 (44.1%)
Prior stroke	107 (14.2%)	88 (11.7%)	82 (10.9%)
Chronic lung disease	336 (44.7%)	324 (43.1%)	291 (38.7%)
Prior PCI	223 (29.7%)	212 (28.2%)	228 (30.4%)
Prior coronary artery bypass grafting	220 (29.3%)	193 (25.7%)	201 (26.8%)
Prior permanent pacemaker insertion or ICD	95 (12.6%)	132 (17.6%)	76 (10.1%)
Preprocedure Hgb	12.37 ​± ​1.92	12.43 ​± ​1.81	12.55 ​± ​1.75
eGFR by MDRD	59.54 ​± ​21.96	58.61 ​± ​21.64	59.67 ​± ​22.07
Preprocedure creatinine, mg/dL	1.27 ​± ​0.89	1.32 ​± ​1.05	1.29 ​± ​0.90
Anticoagulants prescribed within 30 d of discharge	84 (11.2%)	69 (9.2%)	70 (9.3%)
Antiplatelets prescribed within 30 d of discharge	380 (50.5%)	399 (53.1%)	368 (49.0%)
Echocardiographic data			
Days from baseline echo to TAVR	73.37 ​± ​54.11	75.26 ​± ​52.96	74.60 ​± ​57.74
Systolic blood pressure, mmHg	134.59 ​± ​22.95	137.35 ​± ​23.32	139.60 ​± ​23.73
Diastolic blood pressure, mmHg	69.04 ​± ​11.62	68.87 ​± ​12.19	70.15 ​± ​20.92
Heart rate by ECHO, bpm	74.96 ​± ​14.06	72.73 ​± ​13.26	69.16 ​± ​22.11
LVEF, %	55.09 ​± ​12.92	56.41 ​± ​12.31	57.13 ​± ​11.03
AV VTI, m	0.94 ​± ​0.23	0.96 ​± ​0.23	0.98 ​± ​0.23
LVOT VTI, m	0.21 ​± ​0.06	0.22 ​± ​0.06	0.23 ​± ​0.06
Dimensionless valve index	0.23 ​± ​0.07	0.24 ​± ​0.07	0.23 ​± ​0.07
AV area indexed to BSA, cm/m^2^	0.38 ​± ​0.11	0.40 ​± ​0.12	0.40 ​± ​0.10
AV mean gradient, mmHg	38.98 ​± ​14.35	39.39 ​± ​14.51	40.26 ​± ​14.71
AV peak gradient, mmHg	65.65 ​± ​22.59	66.48 ​± ​23.19	68.00 ​± ​23.21
LVOT SVI, mL/m^2^	28.59 ​± ​9.62	29.21 ​± ​10.04	30.82 ​± ​9.93
LA volume index biplane, mL/m^2^	45.27 ​± ​17.61	43.48 ​± ​16.74	44.74 ​± ​15.76
LV end diastolic volume, mL	103.41 ​± ​43.67	102.64 ​± ​43.12	108.34 ​± ​43.16
LV end systolic volume, mL	47.25 ​± ​31.06	45.86 ​± ​32.38	47.41 ​± ​29.58
Aortic regurgitation ≥2+	148 (19.7%)	139 (18.5%)	159 (21.2%)
Mitral regurgitation ≥2+	187 (24.9%)	184 (24.5%)	146 (19.4%)
Annulus size by CT, mm	25.23 ​± ​2.72	25.22 ​± ​2.64	25.31 ​± ​2.65
Arterial access			
Femoral	650 (86.4%)	743 (98.8%)	748 (99.6%)
Subclavian	72 (9.6%)	6 (0.8%)	2 (0.3%)
Aortic	13 (1.7%)	2 (0.3%)	0 (0.0%)
Apical	16 (2.1%)	1 (0.1%)	1 (0.1%)
Caval	1 (0.1%)	0 (0.0%)	0 (0.0%)
SAPIENS3 Valve	662 (88.0%)	655 (87.1%)	622 (82.8%)
SAPIENS3 Ultra valve	89 (11.8%)	97 (12.9%)	129 (17.2%)
SAPIENS3			
20 mm	27 (3.6%)	27 (3.6%)	21 (2.8%)
23 mm	225 (29.9%)	224 (29.8%)	193 (25.7%)
26 mm	240 (31.9%)	233 (31.0%)	226 (30.1%)
29 mm	170 (22.6%)	171 (22.7%)	182 (24.2%)
SAPIENS3 Ultra			
20 mm	5 (0.7%)	7 (0.9%)	12 (1.6%)
23 mm	41 (5.5%)	47 (6.2%)	44 (5.9%)
26 mm	43 (5.7%)	43 (5.7%)	73 (9.7%)
Balloon predilation	136 (18.1%)	149 (19.8%)	113 (15.0%)
Balloon postdilation	263 (35.0%)	319 (42.4%)	316 (42.1%)
Contrast volume, mL	44.73 ​± ​27.92	41.10 ​± ​20.48	43.02 ​± ​25.69
Valve-in-valve TAVR	40 (5.3%)	54 (7.2%)	66 (8.8%)

*Notes.* Values are expressed as either frequencies with proportions for categorical variables or mean ​± ​SD for continuous variables.

Abbreviations: AV, aortic valve; BMI, body mass index; bpm, beats per minute; BSA, body surface area; CT, computed tomography; eGFR, estimated glomerular filtration rate; HR, heart rate; ICD, implantable cardioverter-defibrillator; L/min, liters per minute; LA, left atrial; LVEF, left ventricular ejection fraction; LV, left ventricle; LVOT, left ventricular outflow tract; MDRD, Modification of Diet in Renal Disease; NYHA, New York Heart Association; PCI, percutaneous coronary intervention; STS, Society of Thoracic Surgeons; SVI, stroke volume index; TAVR, transcatheter aortic valve replacement; VTI, velocity time integral.

Age increased slightly across the tertiles, while the proportion of female patients remained similar among the groups. Differences were noted in body measurements, with body mass index gradually decreasing across tertiles. Additionally, diabetes mellitus was more common in the lower tertile and became less frequent in the higher tertiles. Other conditions, such as coronary artery disease, hypertension, and atrial fibrillation, showed no significant differences between groups. Left ventricular ejection fraction (LVEF) increased progressively across the tertiles, as did the indexed aortic valve area and forward stroke volume index, indicating improved cardiac function in the higher tertiles (Table 1).

Femoral access was the most frequently used approach, with its usage increasing across the tertiles, while other access sites were used less often. SAPIEN 3 valves were the most commonly implanted, with no major differences in distribution across groups. Valve size selection did not vary significantly between tertiles. Balloon postdilation was more frequent in the middle and upper tertiles, and the use of cerebral protection devices increased in the higher tertiles (Table 1).

### Association Between HR-Adjusted NWT and Hemodynamic Outcomes

**Figure 3:**
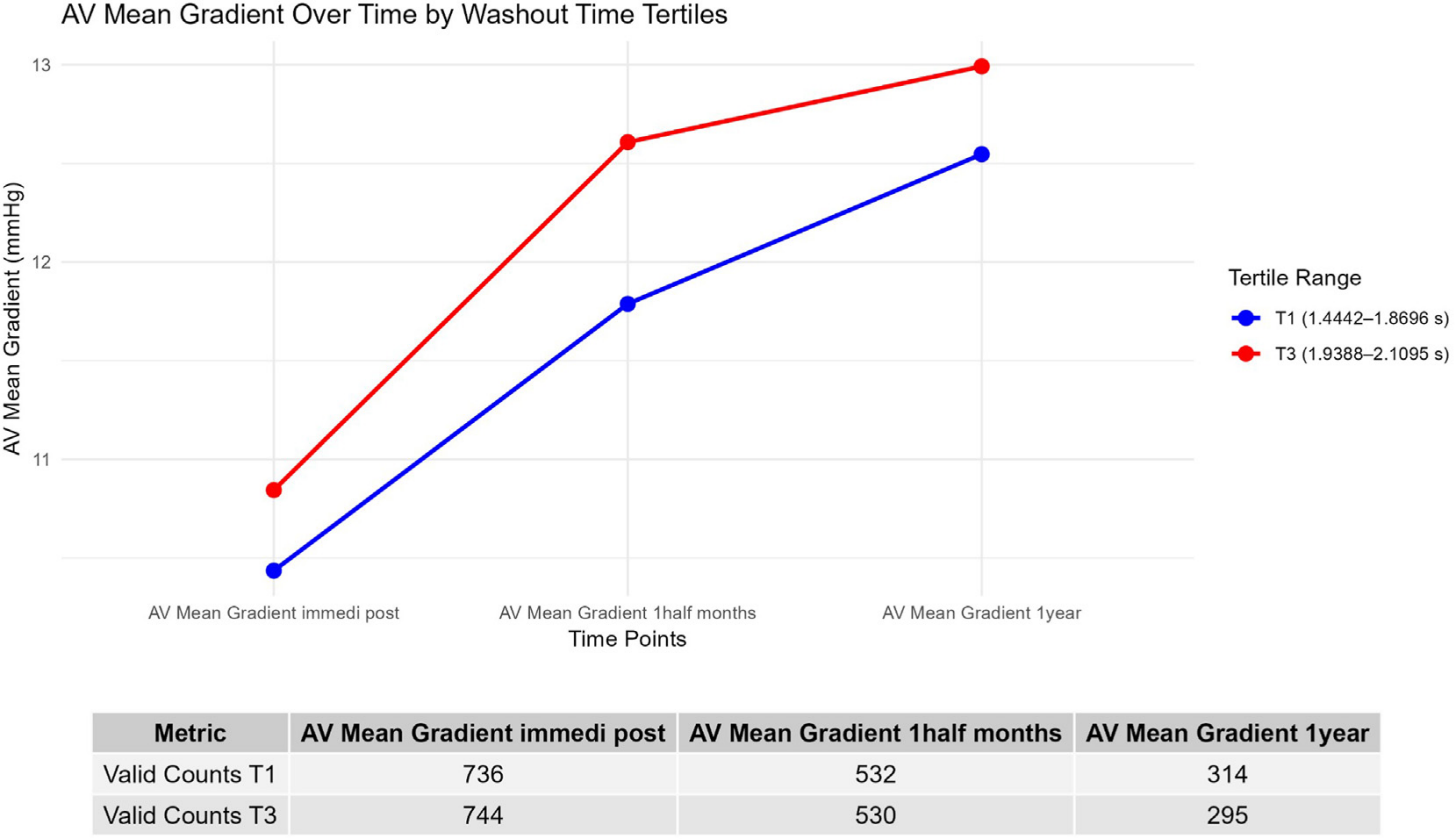
Transvalvular aortic mean gradient over time. Patients with prolonged washout time (tertile 3) had increased mean gradient (in mmHg) at 30 days, but there was no difference at 1 year by *t-*test. Abbreviation: AV, aortic valve.

The median 30-day follow-up time was 47 days (IQR: 37-62) and 1-year follow-up time was 373 days (IQR: 295-427). When considering all valve sizes, at 1-month follow-up, AVMG was higher in T3 compared to T1 (mean ​± ​SD: 12.61 ​± ​5.41 vs. 11.79 ​± ​4.60; *p* ​= ​0.02), as was aortic valve peak gradient (24.13 ​± ​9.85 vs. 22.57 ​± ​8.42; *p* ​= ​0.02). Among the patients with 26 mm valves, at 1-month follow-up, AVMG was higher in T3 compared to T1 (11.72 ​± ​4.99 vs. 10.69 ​± ​3.75; *p* ​= ​0.04), as was aortic valve peak gradient (22.70 ​± ​8.81 vs. 20.63 ​± ​6.94; *p* ​= ​0.02) (Table 2, Figure 3).

**Table 2:** Hemodynamic outcomes stratified by valve size and compared across follow-up times

Characteristics	All valve sizes			20 mm (4%)			23 mm (35%)			26 mm (39%)			29 mm (22%)		
	T1	T3	*p*	T1	T3	*p*	T1	T3	*p*	T1	T3	*p*	T1	T3	*p*
Baseline															
AV mean gradient, mmHg	41.15 ​± ​14.50	41.72 ​± ​14.59	0.1	54.06 ​± ​15.83	47.27 ​± ​12.91	0.33	42.61 ​± ​14.82	41.45 ​± ​14.98	0.98	40.09 ​± ​14.33	41.54 ​± ​14.17	0.30	38.21 ​± ​12.42	41.36 ​± ​14.93	**0.03**
AV peak gradient, mmHg	65.65 ​± ​22.59	68.00 ​± ​23.21	0.1	82.44 ​± ​26.47	74.68 ​± ​17.63	0.39	67.75 ​± ​23.16	68.04 ​± ​23.87	0.99	64.50 ​± ​21.74	67.99 ​± ​22.72	0.17	61.35 ​± ​20.79	66.78 ​± ​23.98	**0.08**
DVI	0.24 ​± ​0.32	0.25 ​± ​0.38	0.1	0.25 ​± ​0.07	0.24 ​± ​0.04	0.38	0.27 ​± ​0.52	0.25 ​± ​0.07	0.64	0.23 ​± ​0.06	0.27 ​± ​0.60	0.36	0.22 ​± ​0.09	0.23 ​± ​0.07	0.5
LVEF, %	54.95 ​± ​13.11	57.04 ​± ​11.00	**0.02**	62.56 ​± ​9.28	59.76 ​± ​9.67	0.46	58.09 ​± ​10.90	60.37 ​± ​9.26	**0.05**	54.56 ​± ​12.62	55.78 ​± ​11.69	0.45	49.30 ​± ​15.33	54.30 ​± ​11.05	**0.001**
Immediate post-TAVR															
AV mean gradient, mmHg	10.44 ​± ​4.53	10.84 ​± ​4.66	0.73	15.76 ​± ​5.4	15.11 ​± ​6.26	0.69	11.73 ​± ​4.89	12.18 ​± ​4.68	0.75	9.64 ​± ​3.76	10.21 ​± ​4.23	0.14	8.72 ​± ​3.52	9.33 ​± ​4.05	0.15
AV peak gradient, mmHg	20.35 ​± ​8.41	20.98 ​± ​8.59	1.00	29.36 ​± ​8.55	27.91 ​± ​11.08	0.60	22.72 ​± ​9.01	23.38 ​± ​8.55	0.54	18.97 ​± ​7.45	19.92 ​± ​7.91	0.25	17.2 ​± ​6.58	18.26 ​± ​7.75	0.2
DVI	0.5 ​± ​0.12	0.51 ​± ​0.12	1.00	0.48 ​± ​0.12	0.47 ​± ​0.15	0.6	0.49 ​± ​0.11	0.5 ​± ​0.12	0.62	0.5 ​± ​0.13	0.52 ​± ​0.12	0.3	0.5 ​± ​0.11	0.51 ​± ​0.12	0.37
LVEF, %	55.52 ​± ​12.29	57.4 ​± ​10.08	**0.003**	63.91 ​± ​7.56	59.93 ​± ​8.4	0.13	58.87 ​± ​10.54	60.22 ​± ​9.11	0.30	54.5 ​± ​11.79	56.34 ​± ​10.23	0.12	50.25 ​± ​13.93	54.93 ​± ​10.44	**0.001**
1-mo follow-up															
AV mean gradient, mmHg	11.79 ​± ​4.6	12.61 ​± ​5.41	**0.02**	15.51 ​± ​4.2	17.03 ​± ​7.36	0.71	13.91 ​± ​4.96	14.4 ​± ​5.79	0.66	10.69 ​± ​3.75	11.72 ​± ​4.99	**0.04**	9.78 ​± ​3.66	10.9 ​± ​3.96	**0.05**
AV peak gradient, mmHg	22.57 ​± ​8.42	24.13 ​± ​9.85	**0.02**	29.64 ​± ​6.45	31.6 ​± ​14.32	0.84	26.31 ​± ​9.16	27.05 ​± ​10.67	0.75	20.63 ​± ​6.94	22.7 ​± ​8.81	**0.02**	18.94 ​± ​6.72	21.22 ​± ​7.64	**0.04**
DVI	0.44 ​± ​0.1	0.45 ​± ​0.1	0.96	0.44 ​± ​0.13	0.43 ​± ​0.12	0.98	0.43 ​± ​0.1	0.44 ​± ​0.1	0.55	0.45 ​± ​0.09	0.45 ​± ​0.1	0.94	0.46 ​± ​0.1	0.45 ​± ​0.1	0.95
LVEF, %	56.32 ​± ​11.13	57.86 ​± ​10.16	**0.05**	62.62 ​± ​7.75	63.16 ​± ​10.15	0.86	58.77 ​± ​9.74	60.89 ​± ​8.76	**0.06**	55.51 ​± ​11.35	56.68 ​± ​9.95	0.44	53.0 ​± ​12.07	54.77 ​± ​10.94	0.23
1-y follow-up															
AV mean gradient, mmHg	12.55 ​± ​5.82	12.99 ​± ​5.55	1.00	17.71 ​± ​10.33	20.93 ​± ​5.15	0.6	14.7 ​± ​5.38	15.35 ​± ​6.57	0.72	11.62 ​± ​5.02	11.61 ​± ​4.23	1.00	9.81 ​± ​5.05	11.17 ​± ​3.93	0.13
AV peak gradient, mmHg	23.83 ​± ​10.63	24.63 ​± ​10.37	1.00	32.41 ​± ​18.76	37.84 ​± ​9.48	0.66	27.9 ​± ​10.02	29.06 ​± ​12.77	0.75	22.23 ​± ​9.07	22.02 ​± ​7.69	0.98	18.67 ​± ​9.07	21.42 ​± ​7.11	0.08
DVI	0.44 ​± ​0.12	0.44 ​± ​0.1	1.00	0.39 ​± ​0.11	0.37 ​± ​0.04	0.8	0.44 ​± ​0.14	0.43 ​± ​0.11	0.72	0.43 ​± ​0.09	0.46 ​± ​0.1	0.05	0.45 ​± ​0.13	0.43 ​± ​0.1	0.59
LVEF, %	55.22 ​± ​11.85	57.87 ​± ​10.07	**0.06**	59.77 ​± ​11.85	61.23 ​± ​9.71	0.94	57.93 ​± ​10.81	60.88 ​± ​9.71	**0.09**	54.71 ​± ​11.39	57.32 ​± ​9.12	0.15	50.68 ​± ​12.95	54.36 ​± ​10.99	0.17

*Notes.* T1 is tertile 1; T3 is tertile 3.

Values are mean ± SD.

*p* values are Bonferroni adjusted for comparison between T1 vs. T3.

Bold values indicate *p* < 0.05.

Abbreviations: AV, aortic valve; DVI, dimensionless valve index; LVEF, left ventricular ejection fraction; TAVR, transcatheter aortic valve replacement.

**Figure 4:**
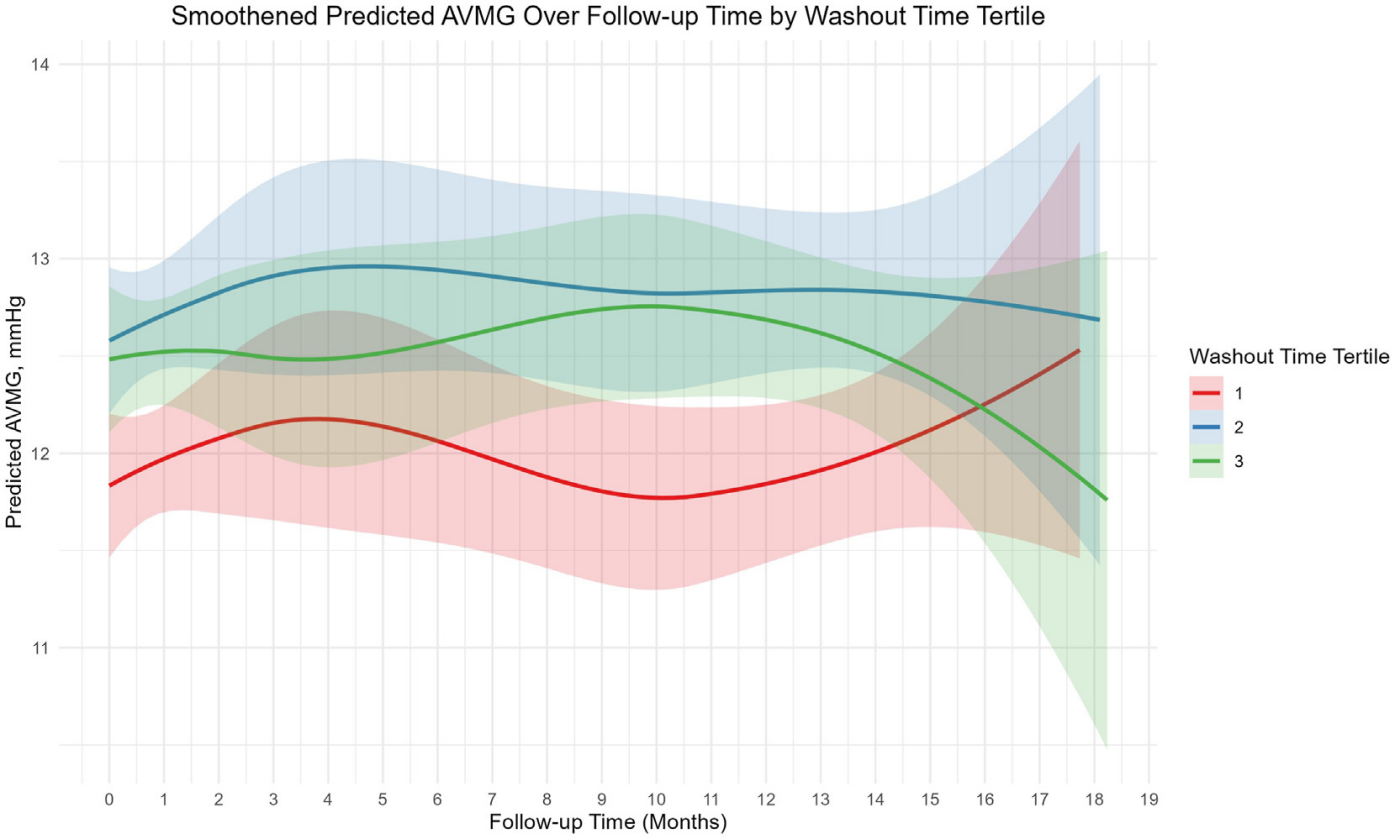
Trajectory of AV mean gradient stratified by washout time over follow-ups. This plot shows the predicted aortic valve mean gradient (AVMG) over 24 months of follow-up, stratified by washout time tertiles (T1, T2, T3) based on a generalized estimating equations (GEE) model. The x-axis represents follow-up time in months, and the y-axis shows predicted AVMG in mmHg. Smooth curves for each tertile indicate trends, with shaded areas representing 95% CIs are obtained by the Locally Estimated Scatterplot Smoothing method. Differences in AVMG trajectories highlight the impact of washout time on valve performance, suggesting that patients with longer washout times (T3) may experience distinct hemodynamic outcomes compared to those with shorter washout times (T1, T2). Abbreviation: AV, aortic valve.

A total of 857 patients who had complete follow-up until 1 year were included in the GEE analysis. In the GEE model, the NWT was adjusted for age, sex, valve size (20 to 29 mm), body mass index, history of atrial fibrillation or flutter, valve type (S3 vs. S3U), valve-in-valve, balloon postdilation done or not, baseline AVMG, baseline LVEF, baseline aortic regurgitation ≥2+, anticoagulant drug prescription, and antiplatelet drug prescription within 30 days of discharge. Across the models, the impact of NWT on outcomes was analyzed. In the GEE model to predict longitudinal DVI changes over time, NWT categorized into tertiles did not show significant associations with DVI values compared to T1 (T2 estimate: −0.009, *p* ​= ​0.52; T3 estimate: −0.012, *p* ​= ​0.45), indicating no significant influence of NWT on this outcome. In the GEE model for AVMG, however, T3 was significantly associated with higher AVMG values follow-up compared to T1 (estimate: 0.703, *p* ​= ​0.02), while T2 showed a nonsignificant trend toward an increase (estimate: 0.498, *p* ​= ​0.12) (Figure 4). In the binary logistic regression model for the combined outcome of an increase in delta AVMG of at least 50% with a decrease in delta DVI of at least 50% (from post-TAVR to 30 days follow-up), NWT modeled using natural spline terms revealed a significant negative association for Spline 1, representing the lower range of washout time values (estimate: −3.71, *p* ​= ​0.04). No significant associations were observed for Spline 2 (estimate: −0.47, *p* ​= ​0.78), Spline 3 (estimate: −3.76, *p* ​= ​0.41), or Spline 4 (estimate: −3.68, *p* ​= ​0.30).

Among 303 patients (13%) who underwent multidetector computed tomography post-TAVR for suspected leaflet thrombosis, 38 (2%) had confirmed HALT. The median time from TAVR to multidetector computed tomography was 385 days (IQR: 70-1043 days). HALT was observed in 13 (<1%) patients at 30 days and 6 (<1%) at 1 ​year. Patients with 30-day HALT had significantly greater NWT (1.9700 ​± ​0.0471 ​s vs. 1.8893 ​± ​0.1001 ​s, z ​= ​−3.37, *p* ​= ​0.001), though differences were not significant at 1 ​year (*p* ​= ​0.51). We tried to find if NWT can be an independent predictor of 30-day HALT by developing and validating a Firth bias-adjusted multivariate logistic regression model. The model was adjusted for age, sex, history of atrial fibrillation or flutter, preprocedural AVMG and baseline LVEF, valve-in-valve procedure, S3 vs. S3U valve, valve size, and anticoagulant or antiplatelet prescription within 30 days of discharge. For every 0.1 ​second increase in washout time, the odds of developing HALT at 30 days are 2.58 times higher (OR: 2.58; 95% CI: 1.11-6.87). To understand the contribution of NWT and HALT to the elevation of AVMG over time, we calculated the variance explained by these factors using pseudo-*R*^2^ values from GEE models. To assess NWT’s impact on AVMG, variance was analyzed using pseudo-*R*^2^ values from GEE models. The baseline model (excluding NWT and HALT) explained 29.8% of AVMG variance. Adding NWT increased this by 0.27%, HALT alone by 0.895%, and both together by 1.15% (total 31.0%). NWT accounted for 28.5% of HALT’s contribution to AVMG elevation, suggesting it may serve as an early marker of valve dysfunction.

## Discussion

We measured NWT in patients who underwent TAVR with balloon-expandable valves at our center. NWT was quantified from post-TAVR aortogram sequences using a machine vision algorithm, revealing a strong association with post-TAVR hemodynamics. After adjusting for HR, NWT was stratified into tertiles: T1 (1.4442-1.8696 ​s; 751 patients), T2 (1.8697-1.9388 ​s; 752 patients), and T3 (1.9388-2.1095 ​s; 751 patients). At the 30-day follow-up, patients in T3 had higher AVMG compared to those in T1 (12.61 vs. 11.98 mmHg). In a multivariate GEE model adjusted for baseline and procedural factors, patients in T3 demonstrated a significant increase in AVMG by 0.703 mmHg compared to T1 (*p* ​= ​0.02), while patients in T2 showed a smaller, nonsignificant increase of 0.498 mmHg compared to T1 (*p* ​= ​0.12). A predictive model for HALT indicated that for every 0.1-second rise in NWT, the odds of developing HALT increased by three-fold. Notably, there is considerable overlap in the mechanisms through which NWT and HALT influence AVMG, with NWT capturing a significant portion of HALT’s effect while independently contributing to gradient elevation.

Given the growing concerns of THV thrombosis, there is a need for earlier identification of treatable risk factors of the same. Although previous studies have quantified the clearance of dye within the neo-sinus in vitro, an in vivo approach that utilizes AI for the same measurement has not been explored before.^7^^,^^16^, ^17^, ^18^ In patients with prolonged washout (T3), an overall increase in the AVMG was observed at the 30-day follow-up post-TAVR. This transient deterioration in the mean gradient at the 30-day follow-up could possibly be multifactorial. The 0.7 mmHg increase in gradients due to T3 of NWT, though adjusted for LVEF, was not adjusted for rhythm or pressure differences during the procedure. Also, differences in observer variability and pressure recovery were not accounted for.^22^ In patients with 29 mm valves and prolonged NWT, higher gradients were noted only at 1 year. Flow stasis, characterized by maximum velocities below 0.05 m/s in the neo-sinus, has been shown to diminish with taller leaflet heights.^23^ In contrast, in vivo data show opposing results, with a larger THV size being a risk factor for THV thrombosis.^1^^,^^8^ This could be due to a possible effect modification due to other thrombogenic factors in vivo. The PARTNER (Placement of Aortic Transcatheter Valve Trial) 3 subanalysis reported 13.3% HALT at 30 days and 27.5% HALT at 1 year, figures that were higher than those observed in our cohort, likely due to the fact that CT was performed only in clinically suspected patients in our cohort.^4^ RELM was observed in all of the patients with HALT. The mere presence of HALT at all time points was not associated with elevated mean gradients.^4^ In a low-risk population, after undergoing TAVR with SAPIEN S3, HALT was present in 16% and hypoattenuation affecting motion in 8% at 30 days.^24^ In another single-center study, which identified 10% of post-TAVR patients at 6 months and 14.3% at 1 year as having HALT, valve hemodynamics and mid-term clinical outcomes were uneventful, even without additional anticoagulant therapy.^8^ The GEE model revealed marginally lower gradients at the 30-day follow-up in patients who were prescribed oral anticoagulants. Although our model included patients regardless of whether HALT was confirmed through imaging, it corroborates the findings from the 3 landmark trials: Global Study Comparing a Rivaroxaban-Based Antithrombotic Strategy to an Antiplatelet-Based Strategy after Transcatheter Aortic Valve Replacement to Optimize Clinical Outcomes (GALILEO), Anti-Thrombotic Strategy to Lower all Cardiovascular and Neurologic Ischemic and Hemorrhagic Events after Transcatheter Aortic Valve Implantation for Aortic Stenosis (ATLANTIS), and Anticoagulation Versus Dual Antiplatelet Therapy for Prevention of Leaflet Thrombosis and Cerebral Embolization After Transcatheter Aortic Valve Implantation (ADAPT). In the GALILEO CT substudy, CT scans performed 3 ​months post-TAVR revealed a significantly lower incidence of RELM in patients receiving rivaroxaban compared to those on dual antiplatelet therapy.^9^ Similarly, the ATLANTIS-4D-CT study demonstrated a notable decrease in RELM or HALT with apixaban over antiplatelet therapy among patients not requiring oral anticoagulant.^25^ The subsequent ADAPT-TAVR trial underscored the efficacy of oral anticoagulant therapy in mitigating the risk of SLT.^26^ Patients in the study who underwent CT imaging 6 ​months after TAVR exhibited a trend toward reduced incidence of leaflet thrombosis when treated with edoxaban compared to dual antiplatelet therapy.^26^ At 30 days, patients with prolonged washout exhibited worsened gradients, though these differences diminished in later follow-ups. Similarly, HALT diagnosed at 30 days resolved over time, consistent with findings from the Low-Risk TAVR (LRT) trial, where early hemodynamic worsening spontaneously improved by 1 ​year.^3^ Patients with elevated gradients and prolonged NWT at 30 days likely experienced spontaneous resolution, as gradients improved despite similar anticoagulant use across tertiles at 1 ​year. NWT was most effective in predicting HALT at 30 days, explaining 28.5% of HALT’s contribution to gradient elevation. This suggests NWT may serve as an early marker of valve deterioration, though other mechanisms remain unaccounted for.

We utilized a novel machine vision algorithm to extract NWT in vivo from routine fluoroscopy images, predicting elevated gradients and HALT occurrence over the course of follow-up. Post-TAVR NWT can serve as a hemodynamic marker of post-TAVR outcomes and help identify at-risk populations. SLT, often missed in transthoracic echocardiography, is most accurately diagnosed with 4D-CT. Routine 4D-CT is not feasible because of radiation and contrast exposure. NWT may be beneficial for personalized anticoagulant pharmacotherapy, potentially aiding in the prevention of future HALT or valve deterioration; however, this hypothesis requires further testing in future studies.

### Limitations

The rhythm and blood pressure during NWT measurement were not measured simultaneously and, therefore, were not accounted for in the analysis. Incomplete opacification of any of the sinus cannot be taken into account by the algorithm and hence remains a limitation. Selection bias for the GEE modeling could potentially limit the applicability, as only complete case analysis was done, thereby also limiting the sample size. The algorithm was originally developed to measure NWT only in balloon-expandable valves and not self-expanding valves. We did not measure THV recoil, which might have explained the elevated gradients observed post-TAVR. Furthermore, we did not measure commissural misalignment, which could affect neo-sinus hemodynamics.^27^ Also, expansion of THV after implantation was not accounted for in the model that was developed to predict HALT. The elevation in gradients predicted by NWT may be statistically significant but not substantial enough to draw clinically meaningful conclusions. Finally, HALT was diagnosed only in patients who underwent CT scans based on clinical suspicion, and the lack of systematic CT follow-up is a major limitation.

## Conclusion

The application of computer vision to angiograms of TAVR patients has revived an intuitive and informative imaging biomarker, the NWT. Prolonged NWT (>1.94 ​s) is predictive of an elevated transvalvular aortic mean gradient upon follow-up post-TAVR and may help to identify patients at risk for 30-day HALT post-TAVR.

## Review Statement

The review of this paper was managed by Guest Editor: James McCabe, MD.

## Declaration of Generative AI and AI-Assisted Technologies in the Writing Process

During the preparation of this work, the author(s) used OpenCV (Python) to collect data from images done during procedure. After using this tool/service, the author(s) reviewed and edited the content as needed and take(s) full responsibility for the content of the publication.

## Ethics Statement

This study represents minimal risk and was approved by the institutional review board of the Cleveland Clinic.

## Disclosure Statement

The authors report no conflict of interest.

## References

[1] Hansson N.C., Grove E.L., Andersen H.R. (2016). Transcatheter aortic valve thrombosis: incidence, predisposing factors, and clinical implications. J Am Coll Cardiol.

[2] Jose J., Sulimov D.S., El-Mawardy M. (2017). Clinical bioprosthetic heart valve thrombosis after transcatheter aortic valve replacement: incidence, characteristics, and treatment outcomes. JACC Cardiovasc Interv.

[3] Khan J.M., Rogers T., Waksman R. (2019). Hemodynamics and subclinical leaflet thrombosis in low-risk patients undergoing transcatheter aortic valve replacement. Circ Cardiovasc Imaging.

[4] Makkar R.R., Blanke P., Leipsic J. (2020). Subclinical leaflet thrombosis in transcatheter and surgical bioprosthetic valves: PARTNER 3 cardiac computed tomography substudy. J Am Coll Cardiol.

[5] Makkar R.R., Fontana G., Jilaihawi H. (2015). Possible subclinical leaflet thrombosis in bioprosthetic aortic valves. N Engl J Med.

[6] Qiu D., Azadani A.N. (2022). On the three-dimensionality of flow in the neo-sinus and its implications for subclinical leaflet thrombosis. Interact Cardiovasc Thorac Surg.

[7] Vahidkhah K., Barakat M., Abbasi M. (2017). Valve thrombosis following transcatheter aortic valve replacement: significance of blood stasis on the leaflets. Eur J Cardiothorac Surg.

[8] Yanagisawa R., Hayashida K., Yamada Y. (2017). Incidence, predictors, and mid-term outcomes of possible leaflet thrombosis after TAVR. JACC Cardiovasc Imaging.

[9] De Backer O., Dangas G.D., Jilaihawi H. (2020). Reduced leaflet motion after transcatheter aortic-valve replacement. N Engl J Med.

[10] Rheude T., Pellegrini C., Stortecky S. (2021). Meta-analysis of bioprosthetic valve thrombosis after transcatheter aortic valve implantation. Am J Cardiol.

[11] Bogyi M., Schernthaner R.E., Loewe C. (2021). Subclinical leaflet thrombosis after transcatheter aortic valve replacement: a meta-analysis. JACC Cardiovasc Interv.

[12] Hatoum H., Dollery J., Lilly S.M., Crestanello J.A., Dasi L.P. (2019). Sinus hemodynamics variation with tilted transcatheter aortic valve deployments. Ann Biomed Eng.

[13] Hatoum H., Lilly S., Maureira P., Crestanello J., Dasi L.P. (2021). The hemodynamics of transcatheter aortic valves in transcatheter aortic valves. J Thorac Cardiovasc Surg.

[14] Hatoum H., Dollery J., Lilly S.M., Crestanello J., Dasi L.P. (2019). Impact of patient-specific morphologies on sinus flow stasis in transcatheter aortic valve replacement: an in vitro study. J Thorac Cardiovasc Surg.

[15] Pott D., Sedaghat A., Schmitz C. (2021). Hemodynamics inside the neo- and native sinus after TAVR: effects of implant depth and cardiac output on flow field and coronary flow. Artif Organs.

[16] Midha P.A., Raghav V., Sharma R. (2017). The fluid mechanics of transcatheter heart valve leaflet thrombosis in the neosinus. Circulation.

[17] Vahidkhah K., Javani S., Abbasi M. (2017). Blood stasis on transcatheter valve leaflets and implications for valve-in-valve leaflet thrombosis. Ann Thorac Surg.

[18] Trusty P.M., Bhat S.S., Sadri V. (2022). The role of flow stasis in transcatheter aortic valve leaflet thrombosis. J Thorac Cardiovasc Surg.

[19] Trusty P.M., Sadri V., Madukauwa-David I.D. (2019). Neosinus flow stasis correlates with thrombus volume post-TAVR: a patient-specific in vitro study. JACC Cardiovasc Interv.

[20] Gollapudi S. (2019).

[21] Kabir M.M., Rahman A., Hasan M.N., Mridha M.F. (2025). Computer vision algorithms in healthcare: recent advancements and future challenges. Comput Biol Med.

[22] Madanat L., Schott J., Mando R. (2023). Discordance vs pressure recovery in aortic stenosis and post-TAVR. JACC Cardiovasc Imaging.

[23] Ncho B., Sadri V., Ortner J., Kollapaneni S., Yoganathan A. (2021). In-vitro assessment of the effects of transcatheter aortic valve leaflet design on neo-sinus geometry and flow. Ann Biomed Eng.

[24] Goel K., Lindman B.R. (2019). Hypoattenuated leaflet thickening after transcatheter aortic valve replacement. Circ Cardiovasc Imaging.

[25] Montalescot G., Redheuil A., Vincent F. (2022). Apixaban and valve thrombosis after transcatheter aortic valve replacement: the ATLANTIS-4D-CT randomized clinical trial substudy. JACC Cardiovasc Interv.

[26] Park D.-W., Ahn J.-M., Kang D.-Y. (2022). Edoxaban versus dual antiplatelet therapy for leaflet thrombosis and cerebral thromboembolism after TAVR: the ADAPT-TAVR randomized clinical trial. Circulation.

[27] Salmonsmith J.A., Ducci A., Burriesci G. (2019). Does transcatheter aortic valve alignment matter?. Open Heart.

