# Supplementary material for: Neo-Sinus Washout Time Following Transcatheter Aortic Valve Replacement and Hemodynamic Outcomes
Source: Struct Heart. 2025 Jun 21;9(9):100686. doi: 10.1016/j.shj.2025.100686 (PMC12395173; doi:10.1016/j.shj.2025.100686)
Supplement: Supplementary material [file mmc1.docx]

**Supplementary material**

**Echocardiographic Evaluation**
All patients in the study received an extensive evaluation through echocardiography, employing standard ultrasonography systems available. Experienced echocardiographers, who were blinded to clinical information, meticulously reviewed and measured all echocardiographic data in accordance with established guidelines.^28^ Echocardiographic parameters included the following variables: Mean (AVMG) and peak transvalvular aortic gradient (AVPG), dimensionless valve index (DVI), Left ventricular ejection fraction (LVEF), LV end-diastolic and systolic volume. AVMG, AVPG, and DVI were measured longitudinally: immediately after the index procedure, and then at 30-days and 1-year follow-up intervals. Transaortic pressure gradient is calculated from velocity using the simplified Bernoulli equation. The DVI is calculated as the ratio of the peak velocity across the valve, measured using continuous-wave Doppler (CW), to the subvalvular velocity obtained via pulsed-wave Doppler (PW).

**Multi Dimensional Computed Tomography evaluation (MDCT)**

Patients who had a clinical suspicion of hypoattenuated leaflet thickening (HALT) underwent MDCT. With complete heart coverage, retrograde electrocardiogram (ECG) gating, and multiphase reconstructions of the aortic root throughout the cardiac cycle, MDCT enabled dynamic four dimensional rendering for the evaluation of valve function. Individual patient dosages of radiation and contrast were optimized, and high spatial resolution was employed. Independent readers evaluated computed tomographic images for HALT using dedicated software (Aquarius iNtuition v4.4, TeraRecon, Foster City, California). The diagnoses were made in accordance with established guidelines.^29^ THV leaflets were evaluated for the presence of HALT using multiplanar reformats aligned with short- and long-axis dimensions.
